# 1,8-Cineole Ameliorates LPS-Induced Vascular Endothelium Dysfunction in Mice via PPAR-γ Dependent Regulation of NF-κB

**DOI:** 10.3389/fphar.2019.00178

**Published:** 2019-03-07

**Authors:** Ke-Gang Linghu, Guo-Ping Wu, Ling-Yun Fu, Hong Yang, Hai-Zhi Li, Yan Chen, Hua Yu, Ling Tao, Xiang-Chun Shen

**Affiliations:** ^1^The Department of Pharmacology of Materia Medica (the State Key Laboratory of Functions and Applications of Medicinal Plants, the High Efficacy Application of Natural Medicinal Resources Engineering Center of Guizhou Province, the Key Laboratory of Optimal Utilization of Natural Medicine Resources), School of Pharmaceutical Sciences, Guizhou Medical University, Guiyang, China; ^2^Institute of Chinese Medical Sciences, State Key Laboratory of Quality Research in Chinese Medicine, University of Macau, Macau, China; ^3^The Department of Pharmaceutics of TCM (the High Educational Key Laboratory of Guizhou Province for Natural Medicinal Pharmacology and Druggability, the Union Key Laboratory of Guiyang City-Guizhou Medical University), School of Pharmaceutical Sciences, Guizhou Medical University, Guiyang, China

**Keywords:** 1, 8-cineole, human umbilical vein endothelial cell, lipopolysaccharide, NF-κB, PPAR-γ

## Abstract

1,8-Cineole (eucalyptol), a monoterpene, has been widely reported for the anti-inflammatory effects. Our previous data confirmed that 1,8-cineole ameliorated the inflammatory phenotype of human umbilical vein endothelial cells (HUVECs) by mediating NF-κB expression *in vitro*. At present, we investigated the protection effects of 1,8-cineole on vascular endothelium in lipopolysaccharide (LPS)-induced acute inflammatory injury mice and the potential mechanisms involved in the protection in HUVECs. Results from enzyme linked immunosorbent assays revealed that 1,8-cineole suppressed the secretion of interleukin (IL)-6 and IL-8 and increased the expression of IL-10 in the serum of LPS-induced mice. 1,8-Cineole reduced the inflammatory infiltration and the expression of vascular cell adhesion molecular 1 (VCAM-1) in the sections of thoracic aorta in LPS-induced acute inflammatory mice. Western blotting indicated that 1,8-cineole significantly decreased the phosphorylation of NF-κB p65 and increased the expression of PPAR-γ in the thoracic aorta tissue. 1,8-Cineole increased the expression of PPAR-γ in LPS-induced HUVECs. 1,8-Cineole and rosiglitazone reduced the protein and mRNA levels of VCAM-1, E-selectin, IL-6, and IL-8 in LPS-induced HUVECs, which could be reversed by the action of GW9662 (inhibitor of PPAR-γ). 1,8-Cineole and rosiglitazone blocked the LPS-induced IκBα degradation and NF-κB p65 nucleus translocation, which could be reversed by the pretreatment of GW9662 or silence of PPAR-γ gene. In conclusion, 1,8-cineole attenuated LPS-induced vascular endothelial cells injury via PPAR-γ dependent modulation of NF-κB.

## Introduction

Cardiovascular diseases (CVDs), emerging from the dysfunction of vascular endothelium, are the leading cause of death worldwide (Mollenhauer et al., 2018). It is well known that inflammation in the pathophysiological process of injured endothelium would deteriorate the damage of endothelial cells and go through the whole process of CVDs (Castellon and Bogdanova, 2016).

Nuclear factor-kappa B (NF-κB) is a transcription factor that regulates expression of many pro-inflammatory proteins, cytokines, chemokines, and adhesion molecules (Sokolova and Naumann, 2017). NF-κB plays a critical role in the regulation of inflammation and various autoimmune diseases (Gambhir et al., 2015). The NF-κB dimers (p65 and p50) bound to its inhibitor (IκB) was found mainly in the cytoplasm in normal cells. Upon activation, IκB is degraded, allowing NF-κB to translocate into the nucleus where it can regulate gene transcription (Rigoglou and Papavassiliou, 2013). Peroxisome proliferator-activated receptor Gama (PPAR-γ) is a member of the nuclear receptor superfamily, PPAR-γ could be activated by ligands, such as endogenous 15-deoxy-D12,14-prostaglandin J2 and insulin-sensitizing thiazolidinediones (Abraki et al., 2013; Ferreira et al., 2014). PPAR-γ appears to be highly expressed during atherosclerotic lesion formation, suggesting that increased PPAR-γ expression may be a vascular compensatory response (Hamblin et al., 2009). Also, ligand-activated PPAR-γ decreased the inflammatory responses in cardiovascular cells, particularly in endothelial cells (Lim et al., 2015). It is also known that PPAR-γ activation leads to the repression of pro-inflammatory genes such as cyclooxygenase 2, inducible nitric oxide synthase and interleukin (IL)-6, and the reduction of cytokines and acute phase proteins by inhibiting the NF-κB pathway (Yang et al., 2014). Ultimately, inhibition of NF-κB by PPAR-γ agonists were reported to reduce the generation of pro-inflammatory mediators/responses (Mingfeng et al., 2014; Marcone et al., 2015; Su et al., 2017).

Lipopolysaccharide (LPS), a endotoxin from the outer membrane of Gram-negative bacteria, causes inflammatory responses in immune and non-immune cells, including endothelial cells (Kim et al., 2014). Absorption of exogenous LPS into blood would lead to the injury of endothelium and increase the secretion of inflammatory cytokines (Li et al., 2017). LPS acts as an critical pathological factor in inflammation by stimulating the secretion of nitric oxide, pro-inflammatory cytokines, chemokines, and adhesion molecules (Ishii et al., 2017).

1,8-Cineole (also known as eucalyptol), a monoterpene found naturally in many aromatic plants, has been widely reported for the bioactivities (Jalilzadeh-Amin and Maham, 2015). 1,8-cineole is one of the main activity compounds of the essential oil from Fructus Alpiniae Zerumbet which is widely used for CVDs in Guizhou China. We had confirmed that 1,8-cineole ameliorated the inflammatory phenotype of human umbilical vein endothelial cells (HUVECs) by mediating NF-κB expression *in vitro* (Linghu et al., 2016). At present, we investigated the protection effects of 1,8-cineole on endothelium of blood vessels in LPS-induced acute inflammatory injury mice and the potential molecular mechanisms in HUVECs.

## Materials and Methods

### Materials and Reagents

1,8-Cineole with a purity >99.5% by gas chromatography analysis was obtained from Aladdin (Shanghai, China). 1,8-Cineole was dissolved in Tween 20 as a stock solution, and diluted with normal saline before intragastric administration to mice. 1,8-Cineole was dissolved in dimethyl sulfoxide (DMSO) as a stock solution, and diluted with endothelial cell medium (ECM) before each cell experiment. Lipopolysaccharide (LPS) from *Escherichia coli* 0127:B8, Rosiglitazone (PPAR-γ agonist) and GW9662 (PPAR-γ inhibitor) were obtained from Sigma-Aldrich (Saint Louis, MO, United States). Human IL-6 (IL-8) ELISA kits and Mouse IL-6 (IL-8, IL-10) ELISA kits were purchased from Neobioscience Technology Co., Ltd. (Shenzhen, China). H&E staining kit, Lysis buffer, Sodium Dodecyl Sulfate-Polyacrylamide Gel Electrophoresis (SDS-PAGE) kit, Enhanced Chemiluminescent (ECL) kit and NF-κB Activation-Nuclear Translocation Assay kit were purchased from Beyotime Biotechnology (Jiangsu, China). PPAR-γ siRNA (GenePharma, Shanghai, China), Lipofectamine 2000 Reagent (Thermo Fisher Scientific, Waltham, MA, United States), RNA Extraction Kit and PrimeScript^TM^ RT Reagent Kit (Takara Bio Inc.) and SsoFast^TM^ EvaGreen Supermix (Bio-Rad, United States) were purchased. PPAR-γ polyclonal antibody (ImmunoWay Biotechnology, Staffordshire, United Kingdom), CD62E antibody (N3C3) and VCAM1/CD106 antibody (N1N2) (GeneTex, Irvine, CA, United States), Goat anti-Rabbit IgG and GAPDH rabbit monoclonal antibody (Bioworld Technology, Nanjing, China), Anti-IκB alpha antibody (EI30) (Abcam, Irvine, CA, United States), and phospho-NF-κB-p65 and glyceraldehyde 3-phosphate dehydrogenase (GAPDH) antibody (Cell Signaling Technology, Danvers, MA, United States) were also purchased. The final concentration of DMSO in the solution was ≤0.1% for all experiments.

### Cell Culture

Human umbilical vein endothelial cell and the ECM were purchased from ScienCell Research Laboratories (San Diego, CA, United States). The ECM was composed of basal medium, 1% endothelial cell growth supplement, 5% fetal bovine serum, and 1% penicillin/streptomycin solution. HUVECs were seeded in cell culture flasks (25 cm^2^; NEST, Shanghai, China) coated with poly-L-lysine, and cultivated in an atmosphere with 95% humidity and 5% CO_2_ at 37°C. Cells were sub-cultured by trypsinization (0.25% trypsin, 0.5 mM EDTA) when they had grown to 80∼90% confluence. Three to six passages cells were used. The culture medium was replaced with serum-free medium for 6 h before treatment with various concentrations of 1,8-cineole with or without LPS (2.5 μg/ml) as indicated for 12 h, and the vehicle control contained serum-free medium only.

### Experimental Animals

Eight-week-old male Kunming mice weighing approximately 20.0–22.0 g were purchased from Guizhou Medical University Laboratory Animal Co., Ltd. (Guiyang, China). They were housed separately under controlled temperature (22 ± 3°C), humidity (50 ± 20%) with an alternating light-dark cycle of 12 h and free access to food and water. All mice were fasted for 2 h before and after drug administration. Thirty-six mice were randomly allocated to six treatment groups as following: Vehicle (0.9% normal saline), LPS (1 mg/ kg), LPS-Dexamethasone (5 mg/kg), and LPS-1,8-Cineole (200,100, and 50 mg/kg), six mice in each group. All drugs were administered intragastrically once daily for 7 consecutive days, and normal saline or LPS was intraperitoneally injected 30 min after drug administration on the last day. After injection with normal saline or LPS for 12 h, blood samples were collected and centrifuged at 3500 rpm for 10 min at 4°C. The serum was collected and stored at -80°C for ELISA assay. All mice were sacrificed and their thoracic aortas were rapidly dissected, some of collected tissues stored in 4% formaldehyde solution, others stored at -80°C. The experimental protocol was approved by Institutional Animal Care and Use Committee of Guizhou Medical University (Guiyang, China), and all procedures were in accordance with the National Institute of Heath’s guidelines regarding the principles of animal care.

### Hematoxylin-Eosin (H&E) Staining

After paraffin embedding, a tissue section with a thickness of 4 μm was prepared in the Department of Pathophysiology, Guizhou Medical University. The tissue slices were heated at 65°C until the wax dissolved and immersed in xylene twice for 5 min each time. Tissue slices were then immersed in 100% alcohol twice, 95% alcohol twice, 80% alcohol once, 70% alcohol once, 50% alcohol once, and washed with running water twice, 2 min each time. The slices were immersed in hematoxylin for 10 min, then rinsed with tap water for 1 min, immersed in 1% hydrochloric acid alcohol for 5 s, and rinsed with lithium carbonate solution for 5 s to turn the slices blue. Next, the slices were immersed in 2% eosin alcohol for 3 min for coloration. 75% alcohol once, 80% alcohol once, 95% alcohol twice, 100% alcohol twice, and in dimethylbenzene twice, each time for 5 min. Finally, neutral gum (Beyotime Biotechnology, Jiangsu, China) was used as a sealant.

### Immunohistochemistry

Paraffin sections were cleared in xylene, rehydrated in graded ethanol (100, 95, 80, and 70%), and subjected to antigen retrieval by microwave (320W, 11 min). Thereafter, the sections were incubated in 3% hydrogen peroxide (H_2_O_2_) for 30 min to abolish endogenous peroxidase activity. The specimens were then blocked for 1 h in 2% bovine serum albumin (BSA), and incubated overnight at 4°C in a humidified chamber with and without rabbit monoclonal antibody against mouse VCAM-1. The samples were then rinsed in phosphate-buffered saline (PBS) and incubated with biotinylated secondary antibody for 30 min at room temperature followed by peroxidase-conjugated streptavidin for 30 min, and developed with 3, 3′-diamino-benzidine. Sections were counterstained with hematoxylin for 1 min. Negative controls were obtained by substituting the primary antibodies with PBS. VCAM-1 positive cells were identified as tan particles in the cell membrane or cytoplasm. The MIAS image analysis system was used to determine the optical density of positive cells. Five visual fields of each sample were randomly selected to measure the average optical density value at 20× magnifications.

### Enzyme-Linked Immunosorbent Assay (ELISA)

Protein levels of IL-6 and IL-8 in supernatants of cultured HUVECs and protein levels of IL-6, IL-8, and IL-10 in serum of LPS-induced mice were detected using an ELISA kit according to manufacturer’s instructions. Absorbance was obtained with a microplate reader (ELx800; General Electric) at 450 nm.

### NF-κB Localization by Immunofluorescence

Detection of NF-κB p65 nuclear translocation was performed as previously (Linghu et al., 2016).

### Western Blotting

Thoracic aorta tissue was lysed with lysis buffer containing 1 mM phenylmethanesulfonyl fluoride (at a rate of 150–250 μL of lysis buffer per 20 mg of tissue and homogenized with a glass homogenizer until the tissue was fully lysed). The lysate was clarified by centrifugation at 14,000 × *g* for 5 min at 4°C. Protein extraction from the HUVECs was carried out as previously (Linghu et al., 2016). The concentration of protein in the supernatant was determined with a BCA Assay kit. Equal amounts (30–50 μg) of protein were separated by 6–12% sodium dodecyl sulfate–polyacrylamide gel electrophoresis and transferred to a polyvinylidene fluoride membrane (Bio-Rad, Hercules, CA, United States). Membranes were blocked using 5% non-fat dry milk with TBST buffer for 1 h, then incubated overnight at 4°C with rabbit anti-mouse p-p65 (1:1000 dilution), rabbit anti-human p-p65 (1:1000 dilution), rabbit anti-human PPAR-γ (1:1000 dilution), rabbit anti-mouse PPAR-γ (1:800 dilution), rabbit anti-human E-selectin (1:1000 dilution), rabbit anti-human VCAM-1 (1:600 dilution), rabbit anti-human IκBα (1:1000 dilution) polyclonal antibodies and GAPDH rabbit monoclonal antibody (1:1000). After washing, membranes were probed further with horseradish peroxidase-conjugated goat anti-rabbit IgG (1:5000). Blots were visualized using an enhanced chemiluminescence kit (Beyotime Institute of Biotechnology). Digital images of blots were produced by a Syngene Gel Imaging System (Bio-Rad) and quantified with Syngene software.

### Quantitative Real-Time RT-PCR

Total RNA was extracted from HUVECs by the TaKaRa MiniBEST universal RNA extraction kit (TaKaRa, Dalian, China) according the manufacturer’s protocol. The content of total RNA was detected by the NanoVue spectrophotometer (Biochrom, United Kingdom). RNA was transcribed to cDNA using the Reverse Transcription Kit (TaKaRa Bio Inc., Kusatsu, Japan) in accordance with the manufacturer’s instruction. Real-time PCR was performed on a Bio-Rad Real-Time PCR System (Applied Biosystems Co., Foster City, United States). The primers were synthesized by Sangon Biotech (Shanghai, China) and sequences were as follows: β-actin, forward 5′-CCTGGCACCCAGCACAAT-3′ and reverse 5′-GGGCCGGACTCGTCATAC-3′; GAPDH, forward 5′-CAGGAGGCATTGCTGATGAT-3′ and reverse 5′-GAAGGCTGGGGCTCATTT-3′; IL-6, forward 5′-GACAGCCACTCACCTCTTCA-3′ and reverse 5′-CCTCTTTGCTGCTTTCACAC-3′; IL-8, forward 5′-GCAGAGGGTTGTGGAGAAGT-3′ and reverse 5′-CCCTACAACAGACCCACACA-3′; E-selectin, forward 5′-TTTGGTGAGGTGTGCTCATT-3′ and reverse 5′-TCTGTCCATTGTCCCTGAGA-3′; VCMA-1, forward 5′-AAGCCGGATCACAGTCAAGTG-3′ and reverse 5′-TCTTGGTTTCCAGGGACTTC-3′. The PCR cycle was performed as follows: stage 1, 1 cycle at 95°C for 30 s; stage 2, 40 cycles at 95°C for 5 s and, 60°C for 30 s. At the end of PCR reactions, melt curve analyses were performed for all genes. The fold increase or decrease was determined relative to a blank control after normalizing to GAPDH and β-actin in each sample using the delta-delta Ct method.

### siRNA Transfection

siRNA (small interfering RNA) targeting PPAR-γ was designed and synthesized by GenePharma (Shanghai, China). For transfection, HUVECs were seeded into 6-well plates (Nest, Biotech, China) at 30–50% confluence. After 24 h, HUVECs were incubated with Lipofectamine 2000 reagent and siRNAs in accordance with the manufacturer’s protocol. The transfected cells were collected 48–72 h later.

### Statistical Analyses

Data are the mean ± SD of at least three independent experiments. ANOVA followed by Dunnett’s test was used to compare three and more groups. The Student’s *t*-test was used to compare two groups. *P* < 0.05 was considered statistically difference.

## Results

### 1,8-Cineole Maintained the Balance of Inflammatory Cytokines in the Serum of LPS-Induced Mice

Endothelial cell is the barrier system between blood and vascular tissue. Pathophysiological change of blood condition would lead to dysfunction of vascular endothelium, in turn, injury of endothelium would lead to blood pathophysiological change (Rodrigues, 2015). To evaluate the protection effects of 1,8-cineole on vascular endothelium, we detected the level of inflammatory cytokines in the serum of LPS-induced mice. As shown in Table 1, LPS (1 mg/kg) induced the increase of pro-inflammatory cytokines (IL-6, IL-8) and decrease of anti-inflammatory cytokine (IL-10) compared with the control group. Pre-treatment with 1,8-cineole and dexamethasone, the secretion of IL-6, IL-8, and IL-10 in the serum were close to normal level. Although 1,8-cineole was tested in a prophylactic manner at present study, we co-incubated the 1,8-cineole and LPS in HUVECs for 12 h *in vitro* and 1,8-cineole protected LPS-induced HUVECs injury, including increased cell viability and the reduced cytokines secretion (Linghu et al., 2016), this indicated that 1,8-cineole would also work in a therapeutic setting.

**Table 1:** 1,8-Cineole maintained the balance of cytokines secretion in the serum of LPS-induced mice.

Group (mg/kg)	IL-6 (pg/ml)	IL-8 (pg/ml)	IL-10 (pg/ml)
Vehicle	50.82 ± 11.68	64.64 ± 5.99	75.14 ± 2.36
LPS (1)	223.88 ± 15.16^##^	481.19 ± 11.50^##^	11.73 ± 2.15^##^
Dexamethasone (5)	34.71 ± 3.61**	183.90 ± 17.50**	20.94 ± 1.92**
1,8-Cineole (50)	157.07 ± 6.01**	270.81 ± 19.47**	23.32 ± 1.92**
1,8-Cineole (100)	50.82 ± 11.68**	183.41 ± 7.70**	25.70 ± 4.77**
1,8-Cineole (200)	9.85 ± 0.24**	90.96 ± 4.90**	57.76 ± 5.71**

*Protein levels of IL-6, IL-8, and IL-10 in serum of LPS-induced mice were determined by an ELISA kit according to manufacturer’s instructions. Data are the mean ± SD (*n* = 6). ^##^*P* < 0.01 vs. vehicle; ^∗∗^*P* < 0.01 vs. LPS group*.

### 1,8-Cineole Reduced the Inflammatory Infiltration to the Artery Wall in LPS-Induced Mice

**Figure 1:**
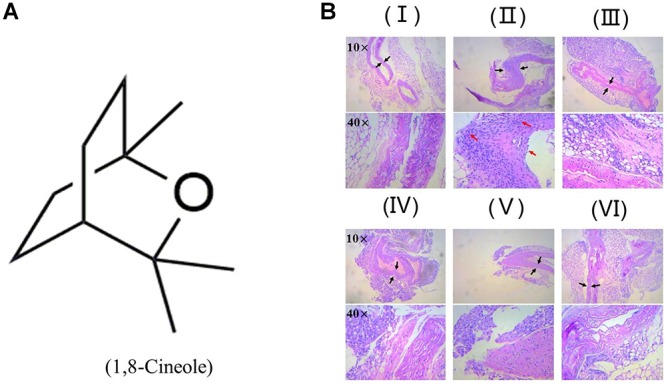
1,8-Cineole reduced the inflammatory infiltration to the artery wall in LPS-induced mice. **(A)** Structure of 1,8-Cineole, which was drawn with ChemDraw software. **(B)** (I) Vehicle, (II) LPS (lipopolysaccharide, 1 mg/kg), (III) Dex (dexamethasone, 5 mg/kg) + LPS, (IV) 1,8-Cineole (50 mg/kg) + LPS, (V) 1,8-Cineole (100 mg/kg) + LPS, (VI) 1,8-Cineole (200 mg/kg) + LPS. After hematoxylin-eosin staining, the pathological images of sections from thoracic aorta were obtained by microscope at 10× and 40× magnifications (*n* = 6). Black arrowhead indicates the swell of artery wall and the red arrowhead indicates the inflammatory infiltration of neutrophil and monocyte to each artery wall layer (intima, media, or adventitia).

Zhao et al. (2014) reported 1,8-cineole reduced the amounts of inflammatory cells in bronchoalveolar lavage fluid, including neutrophils and macrophages. As shown in Figure 1, H&E staining showed the arterial structure in control group was complete and no obvious expansion of vascular lumen and inflammatory infiltration. In LPS group, inflammatory infiltration of neutrophil and monocyte in each artery wall layer (intima, media, or adventitia) was appeared and the vascular wall was swelling. Pre-treatment with 1,8-cineole or dexamethasone, the inflammatory infiltration and swell of artery wall were alleviated significantly.

### 1,8-Cineole Decreased the Expression of VCAM-1 in Thoracic Aorta of LPS-Induced Mice

**Figure 2:**
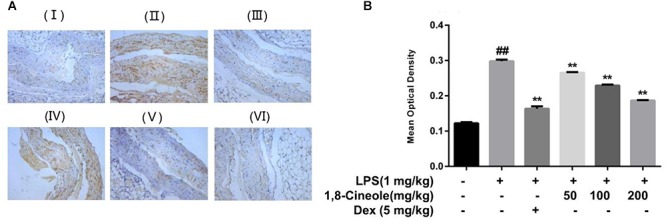
1,8-Cineole decreased the expression of VCAM-1 in thoracic aorta of LPS-induced mice. (I) Vehicle, (II) LPS (lipopolysaccharide, 1 mg/kg), (III) Dex (dexamethasone, 5 mg/kg) + LPS, (IV) 1,8-cineole (50 mg/kg) + LPS, (V) 1,8-cineole (100 mg/kg) + LPS, (VI) 1,8-cineole (200 mg/kg) + LPS. After immunohistochemical staining to VCAM-1, the morphological images **(A)** were obtained by microscope. Five visual fields of each sample were randomly selected to measure the average optical density value at 20× magnifications and make the histogram **(B)**. Vehicle (negative control) was obtained by substituting the primary antibodies with phosphate-buffered saline. VCAM-1 positive cells were identified as tan particles in the cell membrane or cytoplasm. The MIAS image analysis system was used to determine the optical density of positive cells. Data are the mean ± SD (*n* = 6). ^##^*P* < 0.01 vs. vehicle; ^∗∗^*P* < 0.01 vs. LPS group.

VCAM-1 is one of the adhesion molecules expressed on the injured artery wall to mediate the binding of monocytes and lymphocytes to vascular endothelial cells (Wang et al., 2013). We determined the expression of VCAM-1 in thoracic aorta by immunohistochemistry. As shown in Figure 2, compared with control group, the ratio of the VCAM-1 staining in the model group increased significantly, which could be reversed by the pre-treatment of 1,8-cineole or dexamethasone.

### 1,8-Cineole Increased PPAR-γ and Decreased the Phosphorylation of NF-κB p65 in Thoracic Aorta of LPS-Induced Mice

**Figure 3:**
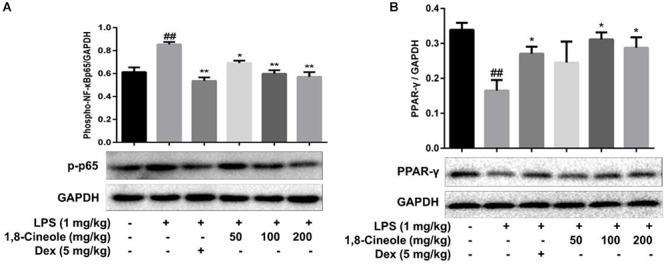
1,8-Cineole increased PPAR-γ and decreased the phosphorylation of NF-κB p65 in thoracic aorta of LPS-induced mice. The expression of PPAR-γ **(A)** and NF-κB p65 **(B)** in thoracic aorta was detected by western blotting. Data are the mean ± SD (*n* = 6). ^##^*P* < 0.01 vs. vehicle; ^∗^*P* < 0.05,^∗∗^*P* < 0.01 vs. LPS group.

PPAR-γ and NF-κB are two key transcription regulators in the process of inflammation (Fu et al., 2014; Su et al., 2017). We detected the expression of these two proteins in thoracic aorta of LPS-induced mice. As Figure 3 shows, LPS induced the decrease of PPAR-γ and increase of p-p65 significantly in model group, these changes were reversed by the pre-treatment of 1,8-cineole or dexamethasone.

### 1,8-Cineole Increased the Expression of PPAR-γ in LPS-Induced HUVECs

**Figure 4:**
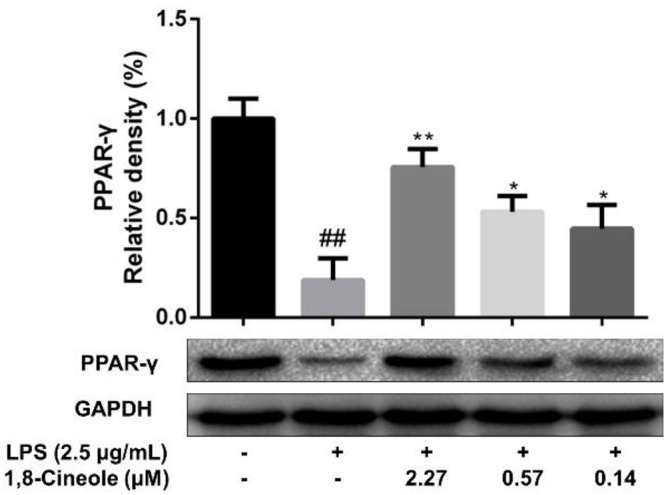
1,8-Cineole increased the expression of PPAR-γ in LPS-induced HUVECs. Cells were incubated with 1,8-cineole (2.27, 0.57, and 0.14 μM) for 1.5 h, then exposed to LPS (2.5 μg/ml) for 12 h. PPAR-γ was examined by western blotting. Data are the mean ± SD (*n* = 3). ^##^*P* < 0.01 vs. control; ^∗^*P* < 0.05, ^∗∗^*P* < 0.01 vs. the LPS group.

To confirm the regulation of 1,8-cineole on PPAR-γ, we examined the expression of PPAR-γ in LPS-induced HUVECs. As shown in Figure 4, LPS induced the reduction of PPAR-γ in HUVECs, which could be ameliorated by pretreatment with 1,8-cineole in concentration-dependent manners.

### 1,8-Cineole Reduced the mRNA and Protein Levels of IL-8, IL-6, E-Selectin, and VCAM-1 in LPS-Induced HUVECs via PPAR-γ Dependent Manners

**Figure 5:**
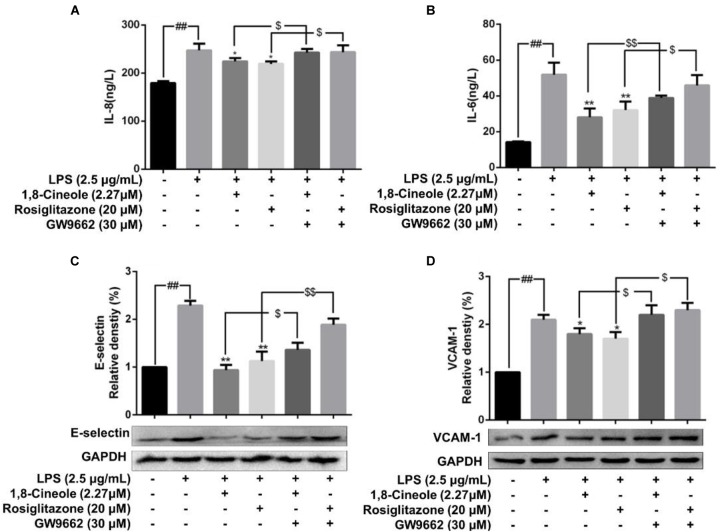
1,8-Cineole reduced the expression of IL-8, IL-6, E-selectin, and VCAM-1 in LPS-induced HUVECs via PPAR-γ dependent manners. Cells were incubated with 1,8-cineole (2.27 μM), rosiglitazone (PPAR-γ agonist, 20 μM), or GW9662 (PPAR-γ inhibitor, 30 μM) for 1.5 h, then exposed to LPS (2.5 μg/ml) for 12 h. Detection of IL-8 **(A)** and IL-6 **(B)** in culture supernatants with ELISA kits. The expression of E-selectin **(C)** and VCAM-1 **(D)** in treated cells was detected by western blotting. Data are the mean ± SD (*n* = 3). ^##^*P* < 0.001 vs. control; ^∗^*P* < 0.05 and ^∗∗^*P* < 0.01 vs. LPS; ^$^*P* < 0.05 and ^$$^*P* < 0.01 vs. GW9662.

**Figure 6:**
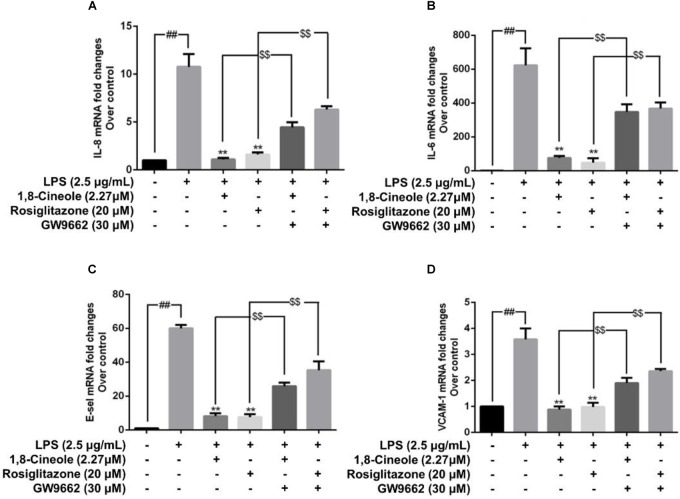
1,8-Cineole reduced the mRNA levels of IL-8, IL-6, E-selectin, and VCAM-1 in LPS-induced HUVECs via PPAR-γ dependent manners. Cells were incubated with 1,8-cineole (2.27 μM), rosiglitazone (PPAR-γ agonist, 20 μM), or GW9662 (PPAR-γ inhibitor, 30 μM) for 1.5 h, then exposed to LPS (2.5 μg/ml) for 12 h. The mRNA expression of IL-8 **(A)**, IL-6 **(B)**, E-selectin **(C)**, and VCAM-1 **(D)** in treated cells was examined by qRT-PCR. Data are the mean ± SD (*n* = 3). ^##^*P* < 0.001 vs. control; ^∗∗^*P* < 0.01 vs. LPS; ^$$^*P* < 0.01 vs. GW9662.

Rosiglitazone, an agonist of PPAR-γ, plays an important role in ameliorating inflammation by activation of PPAR-γ mediating negative regulation of inflammatory signal pathway (Wu et al., 2009). As shown in Figure 5, the expression of pro-inflammatory cytokines (IL-6, IL-8) and adhesion molecules (VCAM-1, E-selectin) were increased significantly in model group. Pretreatment with 1,8-cineole or rosiglitazone could reduce the up-regulated levels of these inflammatory mediators, and these effects could be reversed by the action of GW9662. The similar results for mRNA of IL-8, IL-6, E-selectin, and VCAM-1 were shown in Figure 6.

### 1,8-Cineole Blocked the Activation of NF-κB in LPS-Induced HUVECs via PPAR-γ Dependent Manners

**Figure 7:**
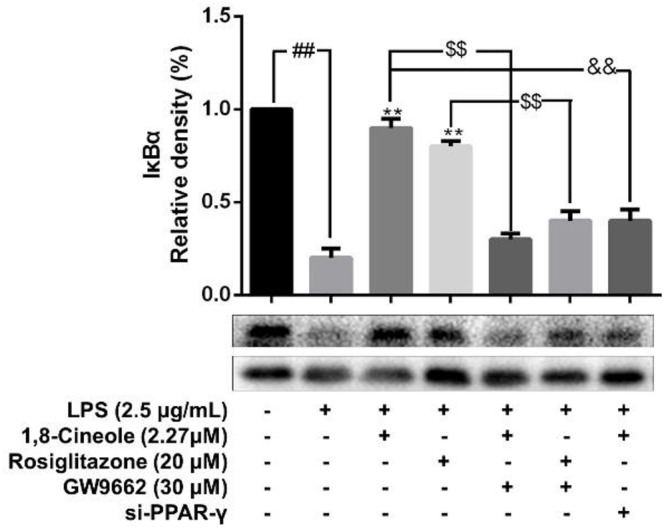
1,8-Cineole inhibited the degradation of IκBα in LPS-induced HUVECs via PPAR-γ dependent manners. Cells with or without si-PPAR-γ (silence of PPAR-γ gene) were incubated with 1,8-cineole (2.27 μM), rosiglitazone (PPAR-γ agonist, 20 μM), or GW9662 (PPAR-γ inhibitor, 30 μM) for 1.5 h, then exposed to LPS (2.5 μg/ml) for 12 h. The expression of IκBα in treated cells was detected by western blotting. Data are the mean ± SD (*n* = 3). ^##^*P* < 0.01 vs. control; ^∗∗^*P* < 0.01 vs. LPS; ^$$^*P* < 0.01 vs. GW9662; ^&&^*P* < 0.01 vs. 1,8-cineole (2.27 μM).

**Figure 8:**
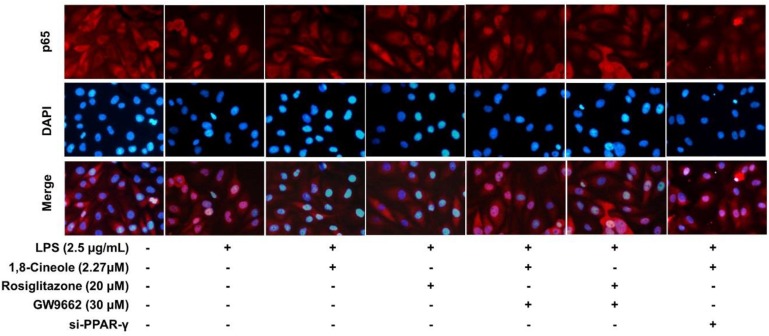
1,8-Cineole attenuated the nucleus translocation of NF-κB p65 in LPS-induced HUVECs via PPAR-γ dependent manners. Cells with or without si-PPAR-γ (silence of PPAR-γ gene) were incubated with 1,8-cineole (2.27 μM), rosiglitazone (PPAR-γ agonist, 20 μM), or GW9662 (PPAR-γ inhibitor, 30 μM) for 1.5 h, then exposed to LPS (2.5 μg/ml) for 12 h. Nuclear translocation of NF-κB p65 was examined by an immunofluorescence staining (×200 magnification) (*n* = 5). Images were obtained using an Nikon fluorescence microscope (nuclei with DAPI staining, blue; NF-κB p65 with Cy3 staining, red). In untreated HUVECs, NF-κB p65 was localized predominantly in the cytoplasm (control group). Cells exposed to LPS (2.5 μg/ml) showed only significant translocation of p65 to the cell nucleus. In cells pretreated with 1,8-cineole (2.27) or rosiglitazone (20 μM) for 1.5 h then exposed to LPS (2.5 μg/ml) for 12 h, NF-κB p65 was retained significantly in the cytoplasm. Cells pretreated with GW9662 (30 μM) or si-PPAR-γ showed significant translocation of p65 to the cell nucleus.

Accumulating data have shown that PPAR-γ acts as upstream of NF-κB in the regulation of anti-inflammatory process, activation of PPARγ plays a negative regulation in inflammatory responses by inhibition of NF-κB (Yenuganti et al., 2014; Bijli et al., 2015). In this part, RNA interference combined with PPAR-γ agonist and inhibitor were used to explore the regulative function of 1,8-cineole on PPAR-γ-NF-κB signal pathway. As shown in Figure 7, 1,8-cineole inhibited the degradation of IκBα, which is similar to the action of rosiglitazone, and these effects could be reversed by the action of GW9662 or silence of PPAR-γ. NF-κB is composed with p65 and IκBα, the degradation of IκBα promotes NF-κB p65 to translocate into the nucleus and initiates transcription of inflammatory factors (Wu et al., 2009). To confirm the inhibitory effects of 1,8-cineole on NF-κB activation, we detected the nucleus translocation of NF-κB p65 with immunofluorescence technique. As shown in Figure 8, cells exposed to LPS showed only significant translocation of p65 to the cell nucleus. In cells pretreated with 1,8-cineole or rosiglitazone for 1.5 h then exposed to LPS for 12 h, NF-κB p65 was retained significantly in the cytoplasm, and this effects were reversed by the action of GW9662 or silence of PPAR-γ gene.

## Discussion

Cardiovascular diseases are well known as the “first killer” to human health (Osuji et al., 2014). Endothelial cells are crucial for maintenance of the physiological functions of the cardiovascular system (Eelen et al., 2015). Endothelial dysfunction (especially inflammation) is closely related to the pathophysiological process of CVDs’ occurrence and development (Castellon and Bogdanova, 2016). Therefore, searching novel therapeutic methods and targets to maintain the homeostasis of vascular endothelial function in the prevention and treatment of CVDs is particularly important.

1,8-Cineole widely exists in volatile oils of aromatic plants (Rocha Caldas et al., 2015). It has been reported that 1,8-cineole presents a wide range of bioactivities including insecticidal (Tak and Isman, 2015), bactericidal (Jardak et al., 2017), hepatoprotective (Murata et al., 2015), and anti-inflammatory (Zhao et al., 2014; Caceres et al., 2017) effects. In our previous study, we had confirmed that 1,8-cineole had a significant protection on LPS-induced vascular endothelial cell injury (Linghu et al., 2016). A further investigation showed that 1,8-cineole-loaded self-microemulsifying drug delivery system could attenuate endothelial injury in mice (Jiang et al., 2019). However, the protection effects of 1,8-cineole on the vascular endothelium injury and the underlying mechanisms have not been completely clarified. In this study, we investigated the protective effects of 1,8-cineole on vascular endothelium in LPS-induced acute inflammatory injury mice, and the potential molecule mechanisms involved in the protection in HUVECs.

Endothelial cells are the barrier systems between blood and vascular tissue. Pathophysiological changes of blood would lead to dysfunction of vascular endothelium, injury of endothelium would further lead to blood pathophysiological changes, forming deteriorated circulation (Rodrigues, 2015). To evaluate the protective effects of 1,8-cineole on vascular endothelium, we detected the levels of inflammatory cytokines in the serum of LPS-induced mice. Based on the efficacy and toxicity from the literatures (Xu et al., 2014; Zhao et al., 2014; Jiang et al., 2019) and our preliminary experiments, we chose dosages of 1,8-cineole (50, 100, and 200 mg/kg) for our investigations. As shown in Table 1, compared with the model group, 1,8-cineole promoted the decrease of pro-inflammatory cytokines (IL-6, IL-8) and increase of anti-inflammatory cytokine (IL-10). Similarly, the inflammatory infiltration (Figure 1) and the expression of VCAM-1 (Figure 2) in the thoracic aorta (intima, media, or adventitia) were significantly reduced in treatment groups. Besides, 1,8-cineole significantly decreased the phosphorylation of NF-κB p65 (Figure 3A) and increased the expression of PPAR-γ (Figure 3B) in thoracic aorta tissue. Totally, the above results suggested that 1,8-cineole protected the vascular endothelium from LPS-induced injury in animal level, maintaining the balance of cytokines secretion, and this action of 1,8-cineole might be attributed to the regulation NF-κB and PPAR-γ. Although 1,8-cineole was tested in a prophylactic manner at present *in vivo* study, we co-incubated the 1,8-cineole and LPS in HUVECs for 12 h *in vitro* and 1,8-cineole protected LPS-induced HUVECs injury (Linghu et al., 2016), this indicated that 1,8-cineole would also work in a therapeutic setting.

NF-κB is a crucial transcription mediator involved in several inflammation-relevant diseases (Park and Hong, 2016). Previously, our results revealed that the protected effects of 1,8-cineole on LPS-induced vascular endothelial cells injury were associated with the reduction of NF-κB-mediated inflammatory cytokines *in vitro*, which was consistent with current experiment results (Figure 3A). PPAR-γ is another crucial transcription factor identified in the inflammatory areas, activation of PPAR-γ plays a negative regulation in inflammatory responses (Shen et al., 2018). As shown in Figure 4B, 1,8-cineole increased the expression of PPAR-γ in LPS-induced HUVECs in a dose-dependent manner, which suggested that 1,8-cineole modulated the expression of PPAR-γ. Subsequently, PPAR-γ agonist (Rosiglitazone, a ligand of PPAR-γ) and PPAR-γ specific inhibitor (GW9662) was used to confirm the regulation of 1,8-cineole on PPAR-γ-medicated inflammatory response. As shown in Figure 5, 6, these results suggested that 1,8-cineole and PPAR-γ agonist had similar effects on inhibition of protein and mRNA levels of VCAM-1, E-selectin, IL-6 and IL-8 induced by LPS, and these effects were reversed by PPAR-γ inhibitor. These results revealed that the activation of PPAR-γ signal was involved in the protective effects of 1,8-cineole on LPS-induced vascular endothelial cells injury.

Increasing evidences have shown that PPAR-γ plays a upstream signal of NF-κB in the anti-inflammatory process (Dammann et al., 2015; Marcone et al., 2015; He et al., 2017). Based on our previous results, further research into investigating protective effects of 1,8-cineole on LPS-induced vascular endothelial cells injury based on PPAR-γ-NF-κB signal pathway is of great value, which is necessary for clarifying the potential molecular mechanism of 1,8-cineole on endothelium protection. Therefore, RNA interference combined with PPAR-γ agonist and inhibitor were used to explore the regulative function of 1,8-cineole on PPAR-γ-NF-κB signal pathway. As shown in Figure 7, 8, the inhibited effects of 1,8-cineole on NF-κB activation (IκBα degradation and nuclear translation of NF-κB p65), which is similar to the action of PPAR-γ ligand (Rosiglitazone), was significantly reversed by the action of specific PPAR-γ inhibitor (GW9662) or siPPAR-γ in HUVECs. These indicated that 1,8-cineole would be a PPAR-γ ligand and suppressed NF-κB activation in a PPAR-γ-dependent manner.

Although, to the best of our knowledge, we did not find any other PPAR-γ-dependent manners were involved in the cascade reaction of inflammation, we could not exclude the possibility that 1,8-cineole ameliorated the inflammation through other PPAR-γ-dependent manners. Besides, it has been reported that PPAR-γ ligands may have anti-inflammatory effects in a PPAR-γ and NF-κB independent manner interfering with other pathways such as activation of ERK (Wilmer et al., 2001) or inhibition of AP-1 pathways (Pérez-Sala et al., 2003), therefore, 1,8-cineole might exert its anti-inflammatory effect even by modulating other pathways.

**Figure 9:**
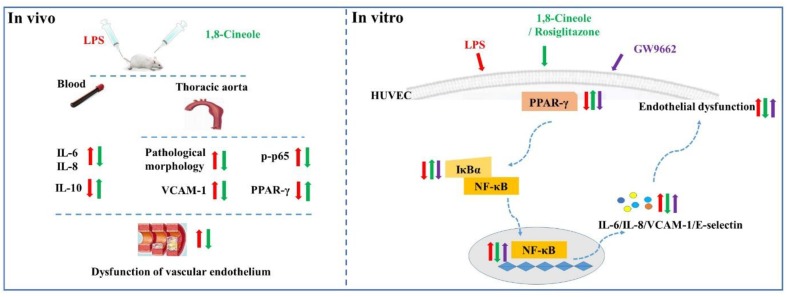
Graphical abstract shows that 1,8-cineole ameliorates the vascular endothelium dysfunction by PPAR-γ dependent regulation of NF-κB *in vitro* and *in vivo*.

In summary, this study evaluated the protection effects of 1,8-cineole on vascular endothelium in animal level, and the relationship among 1,8-cineole, HUVECs and PPAR-γ/NF-κB signal system as shown in Figure 9. Our results show that 1,8-cineole attenuates the LPS-induced vascular endothelium injury by maintaining the balance of pro- and anti-inflammatory cytokines, which involves in the regulation of PPAR-γ and NF-κB. Next, we identified that modulation of 1,8-cineole on NF-κB was PPAR-γ-dependent. Totally, our study suggests that 1,8-cineole would be a potential protection drug for the vascular endothelium exposed to inflammation, and which at least involves in PPAR-γ dependent regulation of NF-κB.

## Author Contributions

K-GL, X-CS, and H-ZL conceived and designed the study. K-GL, G-PW, and H-ZL drafted the manuscript. K-GL, G-PW, L-YF, and HoY performed the experiments and analyzed the data with the help of LT, YC, and HuY. XS and HuY revised the manuscript. All authors read and approved the final manuscript.

## Conflict of Interest Statement

The authors declare that the research was conducted in the absence of any commercial or financial relationships that could be construed as a potential conflict of interest.
